# Effects of sustained natural apophyseal glides versus rocabado 6 × 6 program in subjects with cervicogenic headache

**DOI:** 10.1186/s12891-024-07290-8

**Published:** 2024-02-22

**Authors:** Sadia Murtza, Rabiya Noor, Muhammad Salman Bashir, Mehwish Ikram

**Affiliations:** 1 Faculty of Rehabilitation and Allied Health Sciences, Riphah International University, Lahore, Pakistan; 2 School of Health Sciences, University of Management and Technology, Lahore, Pakistan

**Keywords:** Cervicogenic headache, Mobilization, Pain, Range of motion, Temporomandibular disorders

## Abstract

**Background:**

Cervicogenic headache is designated as the most common type of secondary headache that results from conditions affecting the neck’s bony components, muscles, and intervertebral discs rather than the head itself.

**Objective:**

The purpose was to determine the effects of Sustained Natural Apophyseal Glides (SNAGs) versus the Rocabado 6 × 6 program in subjects with cervicogenic headaches.

**Methods:**

This study was a randomized clinical trial. The sample size was 38, and participants aged 20–60 years (mean age 40.22 ± 9.66) suffering from cervicogenic headaches were randomly allocated using the lottery method into two groups with 19 participants in each group. Assessment of subjects was done before starting treatment and by the end of the 8th week for all the variables. Outcome measures were the Neck Disability Index (NDI), 6-item Headache Impact Test (HIT-6), Flexion-Rotation test (FRT) to assess the rotation range of motion at the level of C1-C2 (goniometer) and the Numeric Pain Rating Scale (NPRS) for the intensity of pain. Data analysis was done by SPSS (IBM) 25. To check the normality of the data the Shapiro-Wilk test was used.

**Results:**

In the Shapiro-Wilk test p-value of all the testing variables i.e. NDI, HIT-6 score, FRT and NPRS was > 0.05, data was normally distributed and parametric tests were used. Group A showed a considerable improvement (*p* < 0.05) in all variables compared to Group B, while within-group analysis of both groups shows that all outcome measures show significant results (*p* < 0.05).

**Conclusion:**

It was concluded that both SNAGs and Rocabado’s 6 × 6 exercises were effective for the treatment of cervicogenic headache but the effects of headache SNAG were superior and produced more improvement in intensity of headache, disability, frequency of headache, duration of headache as compared to Rocabado 6 × 6 exercises.

**Trial registration number:**

This study was registered at ClinicalTrials.gov ID: NCT05865808 on date 19/05/2023.

## Introduction

Cervicogenic headache (CGH) recognized internationally as a distinct clinical entity is a secondary type of disorder resulting from disorders of the cervical region [1]. The concept of headache that originates from the cervical region was introduced by Hilton in 1860, and then by 1983 Sjaastad devised the term cervicogenic headache for such a condition. WHO has declared headaches as one of the top ten threats to human health [2, 3]. Characteristics including location, intensity, frequency, duration and other features of pain are diverse for different types of headache [4]. Characteristics/clinical features of CGH include a one-sided headache that does not move to other sides, with ipsilateral cervical and shoulder pain and neck stiffness affecting the cervical range of motions with worsening of symptoms on neck movement [5]. Cervicogenic headaches are not migraines or tension headaches, sometimes it can be challenging to tell the difference. While there may be some overlap in the symptoms, migraines are mostly brain-based rather than neck-based. Since tension headaches are the outcome of another medical illness, they are also considered primary headaches. Photophobia, phonophobia, vomiting, nausea, and other symptoms of autonomic nature are less common in CGH as compared to other types of headache (migraine) [5, 6]. Patients with CGH commonly show deep neck flexor weakness and tightness of the Sternocleidomastoid (SCM), trapezius and other muscles in the scapular region [6].

A relationship between the neck and mandibular position in Temporomandibular Joint (TMJ) and head postural changes has been reported in many studies [7]. Headache was a much more frequent problem for participants with painful Temporomandibular Disorders (TMDs). This issue requires further research and identification of cause-and-effect relationships. Considering the entire studied population, the relationship between identified TMJ disorders and headache is negligible [8]. Moreover, the higher the degrees of forward head posture and intensity of myofascial pain in neck musculature can elevate the level of cervical muscles myofascial pain and then finally it may lead toward the development of referred pattern of pain in the masticatory musculature and headache [9–12]. The cervical spine, cranium and mandible constitute an anatomical unit known as the “craniocervical-mandibular complex/system”. These neurologic interactions and biomechanical interlay might be the cause of symptoms in the orofacial system, dysfunctions of the cervical region and headaches [10]. The occlusal and skeletal characteristics could have a link with the head posture and painful disorders of the head, jaw, face, neck and cervical spine due to intermingled pathophysiological relationship [12]. The orofacial pain, headache and other symptoms may be associated with impairment of the descending pain modulation pathway, involvement of the second and third branches of the 5th cranial nerve, and central sensitization process [13]. Anatomical interlay between the trigeminal nerve afferent and first 3 spinal nerves on the neurons of the “trigeminal-cervical nucleus” in the upper portion of the spinal cord explains the relation, intermingling of pathway and region of TMJ and neck probably leads to the headache [13–15].

Cervicogenic headache sufferers typically describe complaints of TMD, and individuals with TMDs frequently experience concurrent headaches [16]. It has been discovered that improper masticatory muscle tension is related to head position and is one of the possible reasons for dysfunction in the cervical paravertebral muscles [9, 17]. TMDs has been found involved in several types of headaches including tension, myofascial, and cervicogenic due to the mandibular and temporal joint pathophysiologically. Approximately 44% of patients with cervicogenic headaches experience pain in TMJ, as the two can influence each other it makes sense that TMJ is often involved in people who have cervicogenic headaches [14]. One of the ways TMJ can cause headaches is by changing the distribution of load among various muscles, including those of the head and neck [18]. Headache is one of the common symptoms associated with Temporomandibular Disorders. Both disorders are frequently present in patients, and there is probably a bidirectional relationship between them [19].

Physical therapy including manipulative therapy and therapeutic exercise regimens for the cervical spine and TMJ is very effective in treating a cervicogenic headache [16]. The manual therapy technique activates neural inhibitory systems at different points in the spinal cord and stimulates inhibitory mechanisms [5]. There are various treatment options for CGH like spinal manipulation, and mobilization, massage and dry needling [20]. Biofeedback may be an effective treatment option for patients with different muscle disorders (masticatory muscles) to facilitate normal movement patterns [21]. Low doses of botulinum toxin are effective in the treatment of refractory myofascial pain associated with temporomandibular disorders [22]. Physical therapy, especially Sustained Natural Apopyseal Glides (SNAGs) mobilization by Mulligan, is considered the initial first-line treatment for patients suffering from cervicogenic headaches [23]. “Dr. Mariano Rocabado” developed a 6 × 6 exercise program that consists of six different types of exercises that must be done six times a day with six repetitions of each [24]. Various studies suggest that Rocabado’s technique reduces pain and normalizes the function of the joints of the craniocervical-mandibular system. The Rocabado 6 × 6 approach has been found effective in alleviating aches, restoring the functioning of the masticatory musculature, improving the Forward Head Posture (FHP), improving restricted joint mobility, shortening of muscles, and functional, and postural limitations [25, 26].

The purpose of the current study was to compare the effects of SNAGs and the Rocabado 6 × 6 program in patients with cervicogenic headaches to improve pain, functional limitation and quality of patient’s life. However, there is currently little information available about how physical therapies for TMJ (TMJ-directed approach) reduce the severity of concurrent headaches and improve the quality of life.

## Materials and methods

The study was a randomized clinical trial (parallel group design) and data were collected from Zia Hospital, and Ittefaq Hospital, Pakistan. After obtaining the ethical approval from the institutional ethics committee with a reference number of REC/RCR & AHS/23/0121. This study was registered at ClinicalTrials.gov ID: NCT05865808 on date 19/05/2023. The sample size of 38 was calculated after adding a 10% attrition rate using the epitool sample size calculator with a 5% variance and 95% confidence interval [27]. Using the convenience sampling technique (non-probability), the participants of both genders followed the inclusion criteria of age 20–60 years, participants who had a unilateral headache that did not shift side (ipsilateral neck pain/stiffness), at least once a week in the previous three months, chronic, episodic, moderate to severe pain and duration one hour to weeks (non-throbbing pain starts in the neck). Flexion Rotation Test (FRT) results that are positive with a restriction of more than 10 degrees were randomly allocated into two groups by the lottery method. Each member was approached for the randomization method and then allocated to their respective groups. Participants with a history of other types of headaches, specific disorders and congenital conditions of the cervical spine, receiving Physiotherapy (PT) or chiropractic treatment in the past 3 months or severe pain, neck and head trauma, occlusal splints or any surgery in TMJ area, any history of neurological and cardiovascular disorders, arthritis or patients receiving any medical treatment were excluded from the study. Treatment was provided for 8 weeks and assessment of subjects was done at baseline and the end of the 8th week [16, 28]. The outcome assessor was blinded in this study.

### Group A (headache SNAG)

Patients in this group were treated with the SNAGs. A posteroanterior glide was applied to spinous process of C2 the second cervical vertebrae with ten repetitions held for 10 s during each glide, followed by a 30-second rest period while the patient sitting comfortably and the back supported against an upright chair with relaxed neutral position of the head and neck. Each patient underwent two treatment sessions per week with a maximum of 16 treatment sessions over 8 weeks [16, 23, 28].

### Group B (Rocabado’s 6 × 6 exercises)

Patients were treated with the Rocabado 6 × 6 program which includes 6 exercises that were required to be performed six a day with six repetitions of each exercise [23–26]. Exercises are shown in Table 1. Each patient underwent two treatment sessions per week with a maximum of 16 treatment sessions over 8 weeks.

**Table 1:** Baseline Demographics of Both Groups

Baseline characters	SNAG (Group A)	Rocabado 6 × 6 (Group B)
No. of participants	18	18
Gender	Males = 7 Females = 11	Males = 8 Females = 10
Mean age	40.06 ± 8.93	40.39 ± 10.59

### Outcome measures

#### Neck disability index

The neck disability index (NDI) is used to check the pain intensity in the neck and symptoms of cervicogenic headache. It showed excellent reliability with ICC = 0.92 [29, 30].

### Headache impact test-6 item (HIT-6)

Headache impact test-6 item (HIT-6) was used to investigate the severity of headaches and how they affect functions and social life. The headache impact test showed excellent reliability with ICC = 0.95 [31].

### Flexion rotation test

A Flexion Rotation test (FRT) is used to measure a cervical range of motion employing a goniometer [32]. Flexion rotation test reliability was high with ICC greater than 0.88 [33].

### Numeric pain rating scale

Numeric pain rating scale to assess the pain level of participants [30]. It ranges from zero to ten according to the severity level.

### Statistical analysis

Descriptive and statistical tests were applied using SPSS (IBM version 25). Results of the Shapiro-Wilk test (*p* > 0.05) showed that the data were normally distributed, so parametric tests were applied. The paired t-test shows differences within the group while the differences across the group were shown by the independent t-test and for significant differences, the p-value was set as *p* ≤ 0.05.

## Results

**Fig. 1:**
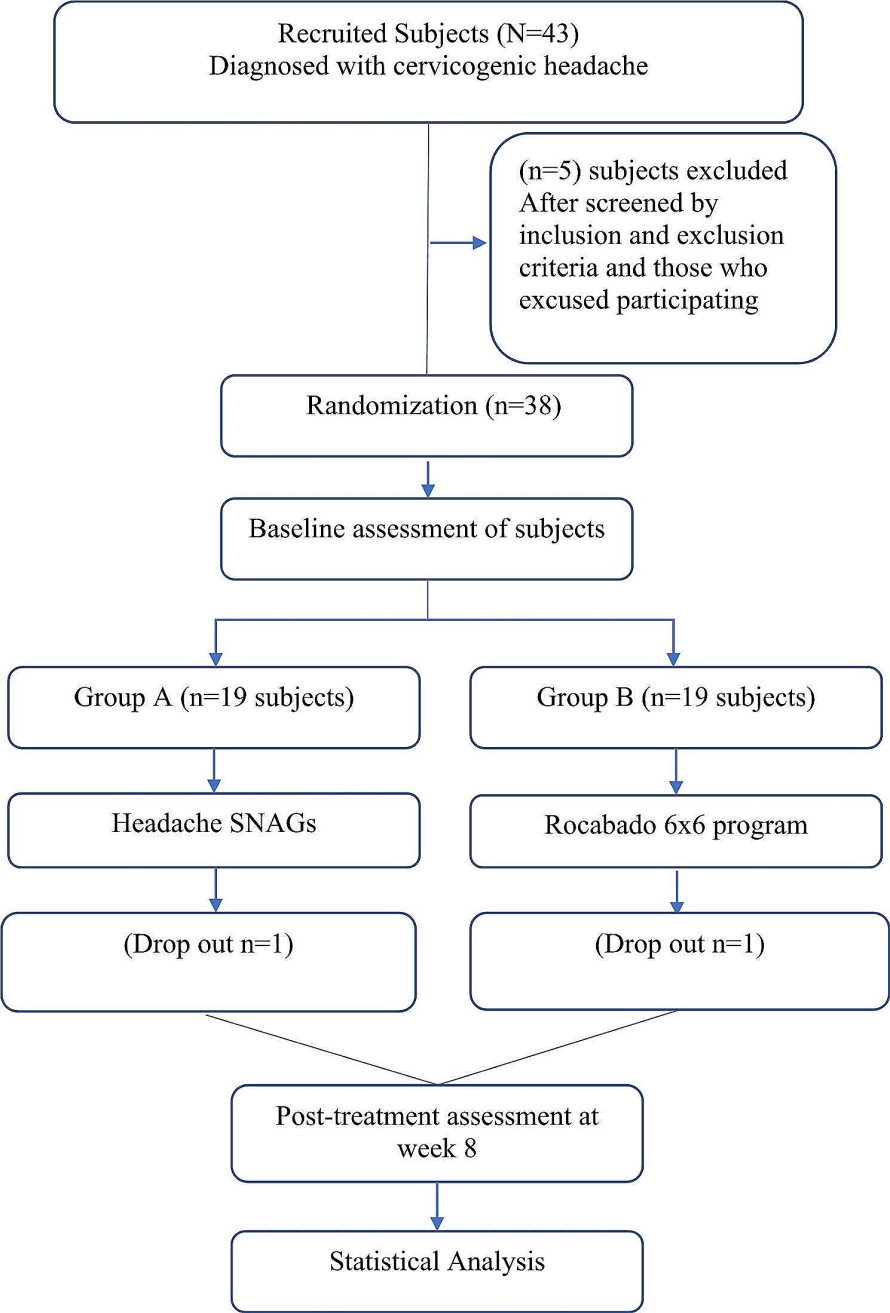
CONSORT Diagram

Out of 43 patients who were screened, 38 people who met the inclusion criteria gave their consent and agreed to take part in the study were recruited for the study. Each patient was asked for their permission after being informed of the study’s safety and their freedom of withdrawal from the study at any time. Before being included, each participant received information about the purpose and methodology of the study. After being accepted into the study and agreeing to participate, participants were allocated into two groups at random using a lottery system. 19 subjects in Group A and 19 in Group B were allocated in both groups using random sampling. 2 participants were dropped off because they were unable to carry the entire treatment regime. Therefore, their data was not analyzed in this study. The flow diagram of the participants is shown in Fig. 1. Pre-treatment demographic data for both groups were compared based on mean ± SD shown in Table 2. The mean age of the participants was 40.17 ± 9.54. There were a total of 21 (58.33%) females and 15 (41.67%) males in the study, they were allocated randomly to Group A and Group B. The data was homogeneous at baseline and no significant difference between groups was shown by independent t-test. The normality of data was tested by using the Shapiro-Wilk test and our data was normally distributed with a p-value of more than 0.05. A parametric test was utilized to compare the various intervals for two populations. Independent t-test was used for between-group analyses and within-group analysis was done by the paired sample t-test.

**Table 2:** Across and within-group comparison of NDI, HIT-6, FRT and NPRS

	Group A (Mean ± S.D)	Group B (Mean ± S.D)	Mean Difference	P-value
Pre-NDI	40.00 ± 6.82	39.72 ± 7.87	0.28	0.911
Post-NDI	10.66 ± 1.81	13.05 ± 2.99	-2.39	0.007
Mean Difference	29.33	26.66		
P-value	0.00	0.00		
	**Group A**	**Group B**	**Mean Difference**	**P-value**
Pre-HIT-6	62.66 ± 7.21	61.66 ± 7.16	1.00	0.679
Post-HIT-6	44.55 ± 3.86	48.83 ± 4.63	-4.28	0.005
Mean Difference	18.11	12.83		
P-value	0.00	0.00		
	**Group A**	**Group B**	**Mean Difference**	**P-value**
Pre-FRT	30.61 ± 3.43	30.72 ± 3.06	-0.11	0.919
Post-FRT	39.72 ± 3.02	36.88 ± 2.44	2.84	0.004
Mean Difference	-9.11	-6.16		
P-value	0.00	0.00		
	**Group A**	**Group B**	**Mean Difference**	**P-value**
Pre-NPRS	6.94 ± 0.93	7.0 ± 0.776	-0.06	0.847
Post-NPRS	2.38 ± 0.91	3.66 ± 1.49	-1.28	0.004
Mean Difference	4.55	3.33		
P-value	0.00	0.00		

[Abbreviations: NDI = Neck Disability Index; HIT-6 = Headache Impact Test 6-item score; FRT = Flexion Rotation Test; NPRS = Numeric Pain Rating Scale]

Table 3 shows the between-group analysis and within-group analysis. Between-group analysis shows that all outcome measures (NDI, HIT-6, FRT and NPRS) show a significant difference (*p* < 0.05) and mean differences show that group A was more effective than group B. In pairwise comparison (paired sample t-test) shows that all outcome measures show significant results (*p* < 0.05) that indicate that both groups show clinical effects.

**Table 3:** Rocabado’s 6 × 6 exercise Program

1-Tongue in the resting position	The patient places the tongue’s tip on the top palate of the mouth, applying light pressure against it
2-TMJ Controlled Rotation	While softly pressing the tongue tip against the palate, open and shut the jaw.
4-Releasing cervical/neck flexion:	To stabilize the entire cervical region, the patient places both hands behind the neck and interlaces the fingers. While the patient conducts flexion, the neck is kept upright. As if nodding his head, raise and drop the chin.
5-Longitudinal extension with Stabilized head	To enhance the functional and mechanical link of the head to the cervical spine, the patient is instructed to stretch his or her head upward while gliding their neck backwards. Push your chin back out after bringing it up towards your neck to form a “double chin”.
6-Retraction of shoulder	The patient is instructed to squeeze the shoulder blades together and draw the shoulders back and down in a single motion alongside lifting and lowering of the chest.

[Abbreviation: TMJ = Temporomandibular Joint]

## Discussion

This research aimed to find out the effects of headache SNAG and Rocabado’s exercises as two different CGH treatments for headache, stiffness and neck pain. Both headache SNAG and Rocabado’s 6 × 6 exercises were found effective for the management of CGH but the effects of the headache SNAG technique were superior and resulted in higher levels of improvements in the intensity of headache, frequency of headache, headache duration and disability. According to literature, long-term effects are usually found with neck exercises while manual therapy provides short-term effects, both can be beneficial for cervicogenic headaches. However, we need more high-quality studies or evidence-based studies on this topic in future to evaluate concurrent conclusions [34]. Many factors influence headaches and can be misdiagnosed with the original cause as dental caries, sleep bruxism, and TMD can cause headaches. Orofacial pain syndromes and headaches are more common in people (mostly in females) with depression which can further lead to sleep disturbances. The most important risk factors for headaches include the overuse of migraine medications, less educated about quality of life, obesity, depression/anxiety, stressful events and aging [34–37].

A study was conducted on the effectiveness of different physiotherapy interventions in the management of cervicogenic headaches by Monika Rani and Jaspreet Kaur in 2022. This study compared the effectiveness of postural correction exercises with SNAG mobilization. Significant improvement in included variables was shown by both techniques compared to the control group. The age range of participants in this study was the same as in the current study which was 20–60 years and the same parameters were used. The findings of the current study were in line with this study and SNAG mobilization showed better [38]. The current research results of cervical SNAG mobilizations were in accordance with those from an earlier study by Ricardo Cardoso et al. in 2022 that assessed the impact of SNAG mobilization on the Flexion Rotation Test, pain intensity, and functionality in subjects with CGH. It was a systematic review of randomized trials. The Flexion Rotation Test (FRT), NPRS, Visual Analogue Scale (VAS), Headache Disability Inventory (HDI), Dizziness Handicap Inventory (DHI), and the Neck Disability Index (NDI) were outcome variables used to evaluate function. The results of this review indicate that SNAG improved headache, FRT, and pain intensity-related functionality. The age range of the current study was comparable to Cardoso’s systematic review. All other variables NDI, HIT-6, FRT and NPRS in the current study showed significant improvement similar to comparative research [6].

Manzoor and his colleagues 2021 studied the effect of SNAGs and strengthening exercises of the cervico-scapular in improving range of motion for the cervical region and reducing pain levels in cervicogenic headache patients. The SNAG mobilization group reported superior results in the study. Pain intensity decreased to a greater extent in the SNAG group as compared to the cervico-scapular strengthening group. There was a greater reduction in the NDI of the SNAG group as compared to the cervico-scapular strengthening group. The results of this study support current study findings [39]. Effects of physical therapy for temporomandibular disorders on headache pain intensity, a systematic review conducted by Hedwig A. van der Meer in 2020. This study aimed to evaluate the literature systematically on the effectiveness of physical therapy for TMD on concomitant headache pain intensity. The therapies varied across the five included articles. The certainty for the effectiveness of physical therapy for TMD on headache intensity was found low. It was concluded that TMJ-directed physical therapy interventions presented a small effect on reducing headache pain intensity in patients with headaches. These findings were in opposition to the current study’s results as Rocabado (TMJ-directed technique) produced significant improvement of all included variables for treatment of cervicogenic headache [16].

Prior research in 2019 by Pahinian et al., however, showed the opposite outcomes when comparing the effects of SNAGs with craniosacral therapy on cervico-genic dizziness. The 30 individuals in the trial were randomly assigned to two groups, cranial-sacral therapy and SNAG. It was found that craniosacral therapy had more significant effects than SNAG while in the current study, SNAG showed superior results in relation to comparative exercise treatment protocol [40]. Studies have proved that Headache SNAG (target C2) specifically is more effective than all other types of Mulligan Mobilizations, that’s why its effectiveness has been compared with other types of therapies. In the majority of literature, SNAGs are more specific and focused on the cervical region only while Rocabado exercises cover a wider area including the craniocervical-mandibular system. In this study as there was no specific criteria was followed either patients were suffered from cervical region disorder or TMD. Clinicians should consider the use of Rocabado’s 6 × 6 exercises for individuals with CGH as a safe and effective alternation of mobilization in patients for whom mobilization is contraindicated.

The limitations of this study were a relative subjectivity to the scales that could have been influenced by the individuals’ psychological support, mood, level of understanding and personalities. Additionally, factors other than the length of treatment, particularly the participant’s drug (medication) usage, could not be properly controlled. No follow-up was conducted in this study and long-term effects were not evaluated. Inclusion criteria did not specifically rule out specific cervicogenic headache patients from those who have TMD.

## Conclusion

It was concluded that both SNAGs and Rocabado’s 6 × 6 exercises were effective for the treatment of subjects suffering from CGH but the SNAGs showed more improvement in reducing intensity of headache, disability, frequency of headache, duration of headache as compared to Rocabado 6 × 6 program.

## Data Availability

Data will be available at a reasonable request from the corresponding author.

## Abbreviations

SNAGs: Sustained Natural Apophyseal Glides
CGH: Cervicogenic Headache
TMD: Temporomandibular disorder
IHDS: International Headache Society
ICHD: International Classification of Headache Disorders
NDI: Neck Disability Index
HIT-6: Headache Impact Test 6-Item Score
FRT: Flexion Rotation Test
NPRS: Numeric Pain Rating Scale
CONSORT: Consolidated Standards of Reporting Trials
